# On the hunt for candidate genes involved in host-adaptation in *Pseudomonas aeruginosa* using comparative genomics

**DOI:** 10.1186/s12864-026-13405-3

**Published:** 2026-10-03

**Authors:** Agata Marchi, Marcus Wenne, Vi Varga, Johan Bengtsson-Palme

**Affiliations:** 1 Division of Systems and Synthetic Biology, Department of Life Sciences, SciLifeLab, Chalmers University of Technology, Gothenburg, SE-412 96 Sweden; 2 Centre for Antibiotic Resistance Research in Gothenburg (CARe), Gothenburg, Sweden; 3 Department of Infectious Diseases, Institute of Biomedicine, The Sahlgrenska Academy, University of Gothenburg, Gothenburg, Sweden

**Keywords:** Genomic dark matter, Host colonization, Opportunistic pathogens, Pathogenicity, *Pseudomonas aeruginosa*

## Abstract

**Background:**

*Pseudomonas aeruginosa* is an opportunistic pathogen responsible for causing severe hospital-acquired infections, largely due to its adaptability, diverse range of virulence factors and drug resistance mechanisms. Despite extensive research, a substantial portion of the genome of *P. aeruginosa* remains functionally uncharacterized. The aim of this study was to identify candidate host-adaptation genes, including potential virulence factors, among these numerous uncharacterized genes.

**Results:**

We performed a comparative genomic analysis of *P. aeruginosa* strains isolated from human clinical sources versus those from natural environments to identify clusters of homologous proteins significantly enriched with sequences from bacteria isolated from humans. Our analysis revealed that approximately 3% of all protein clusters showed significant enrichment in sequences from human isolates. Functional characterization of 45 selected clusters with particularly large enrichment of human isolates showed that these clusters contained genes with roles primarily related to host interaction and horizontal gene transfer. Notably, twelve of the 45 selected clusters consisted of genes with unknown function, suggesting the existence of potentially novel virulence factors or genes involved in the infection process within human-derived isolates.

**Conclusions:**

Our findings highlight specific uncharacterized protein clusters as promising targets for future investigation. The identification of both known virulence-associated functions and uncharacterized proteins among human-enriched clusters demonstrates the ability of our comparative genomics approach to detect established as well as potentially novel candidate host-adaptation genes. These findings further suggest that this strategy may be broadly applicable to the study of other opportunistic pathogens.

**Supplementary Information:**

The online version contains supplementary material available at https://doi.org/10.1186/s12864-026-13405-3.

## Background

Opportunistic pathogens are a prime example of adaptability, as these microorganisms typically exist harmlessly in their host or preferred environments but have the ability to cause infections under certain conditions. The shift to a pathogenic state is often triggered by disruptions in the host's defense mechanisms, such as illnesses or wounds, disturbances to the normal microbiota, or a weakened immune system [5].

*Pseudomonas aeruginosa* is a Gram-negative opportunistic bacterial pathogen commonly found in natural environments like soil and water, but also in healthcare settings where it is a leading cause of nosocomial infections, primarily targeting the airways [21]. This is largely due to its high degree of resistance to many antibiotics, which make *P. aeruginosa* infections particularly challenging to treat, as well as its extensive arsenal of virulence factors – the gene products used to evade the host’s immune defenses, establish infection and cause damage to host tissues. The main virulence factors of *P. aeruginosa* include structural components like lipopolysaccharide and outer membrane proteins, which protect the bacterium and aid in antibiotic resistance,secretion systems and toxins which damage host tissues and impair immune responses; exopolysaccharides that contribute to biofilm formation, enhancing the bacteria’s persistence and resistance to antibiotics; siderophores for iron acquisition and antioxidant enzymes for protection against oxidative stress [20]. All these components play crucial roles in enhancing *P. aeruginosa*'s ability to survive in the host environment and cause infections.

When the complete genome of *P. aeruginosa* PAO1 strain (NCBI:txid208964) was first published, 45.8% of the open reading frames corresponded to genes with unknown functions [5]. Despite extensive research efforts in the years since, approximately 36% of the 5,713 annotated genes in the *P. aeruginosa* PAO1 reference strain genome still code for hypothetical proteins. This significant knowledge gap not only emphasizes the need for continued research but also suggests potential for discovering novel virulence factors that could illuminate *P. aeruginosa's* mechanisms of pathogenicity.

Comparative genomics offers a promising approach to address this [13, 22], particularly through the analysis of strains isolated from different sources [16]. For example, Freschi et al. [7] analyzed over 1,300 *P. aeruginosa*, discovering a very small core genome (1% of the total genes) with an extensive accessory genome shaped by horizontal gene transfer, largely responsible for the pathogenicity of the pathogen. Similar results were obtained by Berger et al. [4], who compared environmental and clinical isolates of *P. aeruginosa* linking virulence traits to specific genomic islands in the accessory genome. Furthermore, Weimann et al. [31] analyzed almost 10,000 *P. aeruginosa* isolates from a variety of sources and studied horizontal gene transfer events and mutational adaptations to host-associated lifestyles, allowing the creation of an evolutionary map of how specific clones can come to dominate in human-associated settings. These clones also appeared to employ different strategies for infection of cystic fibrosis lungs, including adaptation to survive inside macrophages. By using mutation analysis to identify host-adapted genes, that study also revealed convergent, host-specific adaptation, and by unraveling the recent mutational history of epidemic clones, 224 genes that had a higher ‘mutational burden’ were identified, with many of those genes encoding functions related to macrophage survival and host interaction.

**Fig. 1:**
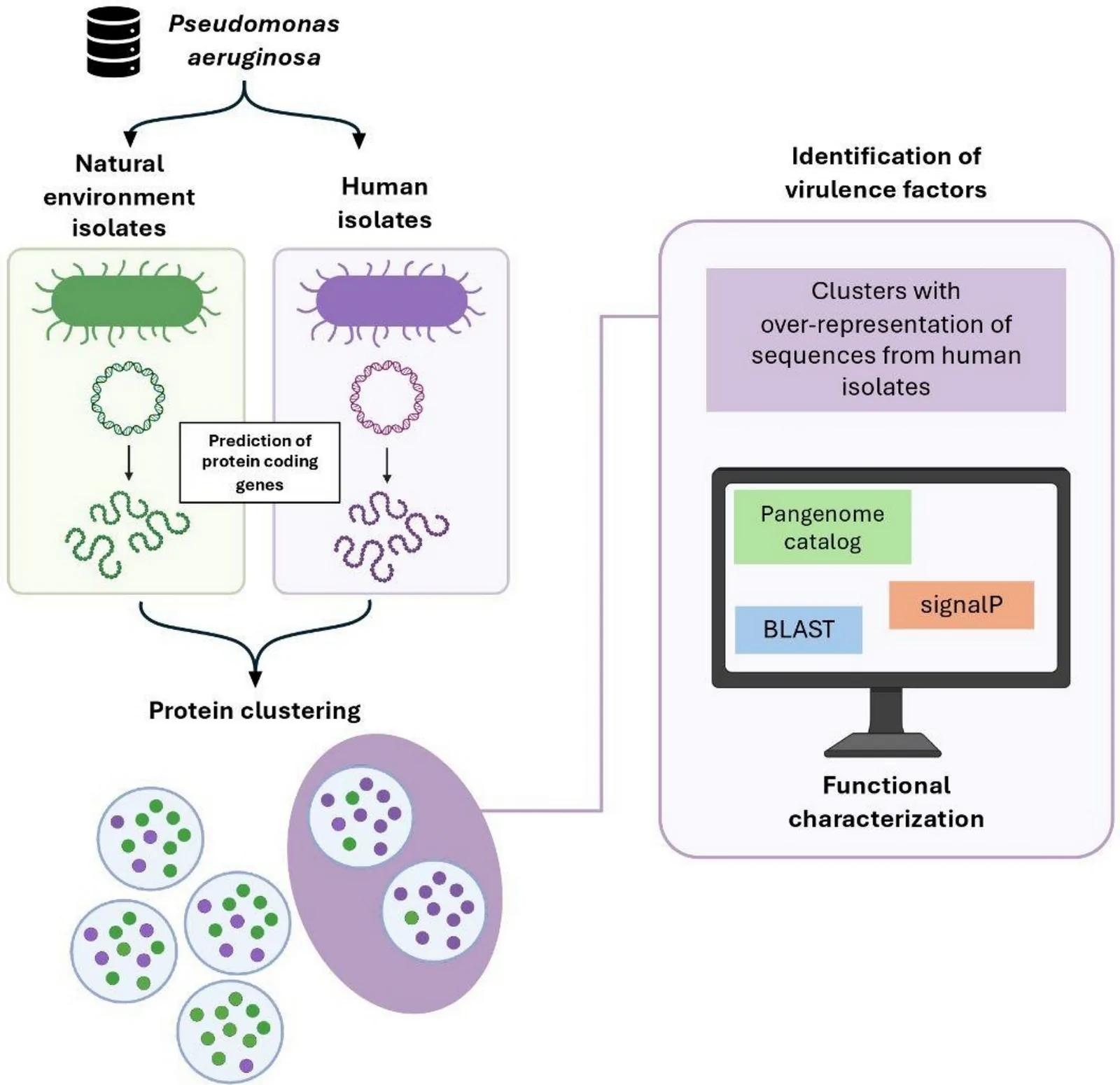
Study overview. NCBI Assembly database and classified as human-associated or environmental based on metadata annotation. Predicted protein sequences were clustered at 90% amino acid identity, and clusters with significant overrepresentation in human isolates were identified by statistical testing. Forty-five representative sequences from enriched clusters were selected for functional characterization using a combination of sequence, structural, and comparative genomic tools

In this study (Fig. 1), we similarly leveraged publicly available genome assemblies of *P. aeruginosa* to compare isolates from natural environmental sources with those isolated from human hosts, aiming to investigate uncharacterized genes and identify potential novel host-adaptation genes and virulence factors. We hypothesize that genes that are enriched among human-associated isolates are more likely to be involved in host colonization and potentially virulence. Through this analysis, we sought to shed light on the genetic basis of *P. aeruginosa*'s pathogenicity using a complementary method, specifically focusing on the uncharacterized portion of the bacterial genome. This work could ultimately contribute to the development of more effective strategies to combat infections caused by this opportunistic pathogen.

While the most relevant genomic comparisons may vary depending on the available data and research context, this methodology can be extended to other less-studied pathogens beyond *P. aeruginosa.* To ensure broad applicability, we developed a flexible bioinformatics approach that incorporates comprehensive isolation source data spanning multiple categories beyond those analyzed in this study and allows for easy modifications and additions to isolation categories, facilitating the adaptation of our methodology to diverse research scenarios.

## Methods

### Retrieval, filtering, and classification of P. aeruginosa genome assemblies

We downloaded all available *P. aeruginosa* genome assemblies and associated metadata from the National Center for Biotechnology Information (NCBI) assemblies database [23] on April 16th, 2024. Using the NCBI datasets command line tool (v16.8.1) [18], we retrieved 41,874 genome assemblies, which comprised the entire collection accessible in the database at that date (Table S1).

For each genome, the host and isolation source terms were extracted from the metadata and processed to classify the genome by assigning to it one of the following labels:

- Human: Settings directly associated with a human host.
- Natural Environment: Settings not directly associated with a human or animal host and not located in an indoor building where humans normally reside (e.g. soil, water).
- Built Environment: Settings located in an indoor building where humans normally reside.
- Other: Settings that do not match any of the above categories, mostly consisting of plants and animals.
- Unknown: missing or unclear metadata.

**Fig. 2:**
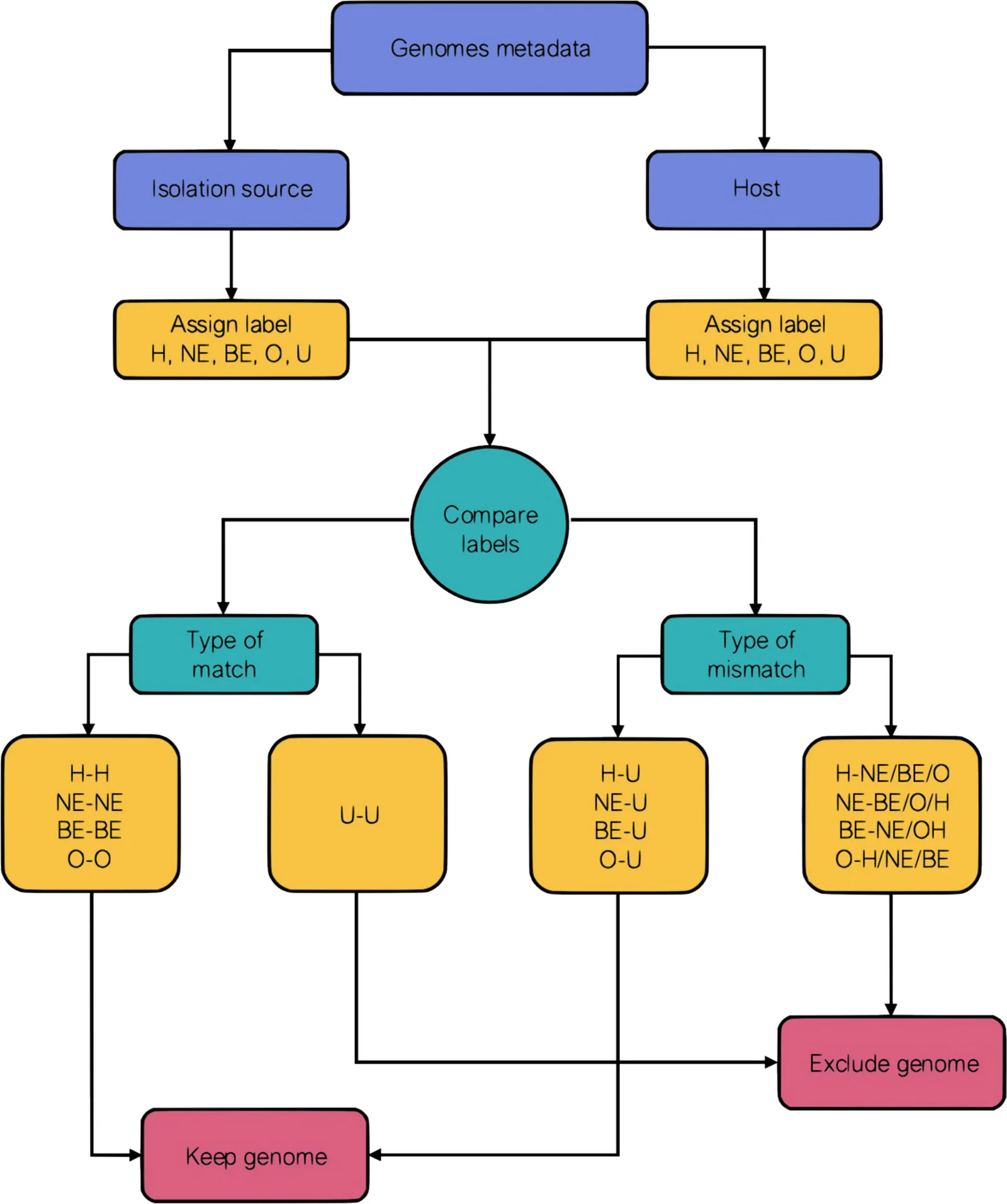
Classification of the genomes. From each genome’s metadata we retrieved the information relative to the host and the isolation source and assigned them a label (H: human, NE: natural environment, BE: built environment, O: other, U: unknown). For each genome, the host and isolation source labels were compared to check if they matched or not. Based on the type of match/mismatch, the genome was kept or discarded

The script was designed to perform this task automatically for obvious cases, while it allowed manual inspection and classification of more complicated entries. Each genome was then classified based on the combination of its host and isolation source labels: if the two labels matched, the genome was assigned that label, but if one label was unknown, the genome was assigned the other label. Assemblies with conflicting labels or with both host and isolation source labeled as unknown were discarded (Fig. 2).

Next, assemblies smaller than 5 Mbp or larger than 8 Mbp were removed from the dataset. As the size of the *Pseudomonas aeruginosa* PAO1 reference genome (NCBI:txid208964, genome assembly ASM676v1) is approximately 6.3 Mbp, assemblies significantly smaller or larger than this were considered likely to represent incomplete or contaminated assemblies and were therefore removed from the analysis. We used MetaxaQR v3.0 [3] to cross-check that none of the genomes included in the human or natural environment subsets were derived from non-Pseudomonas bacterial species.

For the human isolates, additional criteria were applied. Only genomes with host descriptions containing the terms 'human', 'homo sapiens', 'male', ‘female’, ‘man’, ‘woman’ or 'patient', and isolation source descriptions containing the terms 'wound', 'swab', 'urine', 'infection', 'ulcer', 'trachea', 'lung', 'tissue', 'throat', 'sputum', 'stool', 'bronchus', 'cystic fibrosis', 'cf', or 'blood' were retained. Finally, to address the overrepresentation of genomes derived from human isolates in the dataset, this group was randomly subsampled so that its size matched the combined size of the three other groups. This procedure balanced the sizes of the classes in the dataset and the additional selection criteria increased our confidence that these genomes were truly human-associated.

To ensure that a lack of clonal diversity would not bias the analysis, we used skani (v0.3.0) to estimate average nucleotide identity (ANI) between all pairs of genomes in the subsampled dataset [25]. From this analysis, we would expect that if there were major clonal biases, those would result in peaks at 100% or close to 100% identity in the ANI comparisons (Supplementary Figure S1). However, this analysis revealed no overrepresentation of close to identical pairs of genomes for the human-associated strains in comparison to any of the other groups. Instead, the genomes show a distribution mostly in the 98% to 99.5% range, which is what would be expected if the genome population is diverse but that the genomes belong to the same species. The only subsets that showed peaks close to 100% were the ‘Built environment’ and ‘Other’ groups. However, those two datasets were not used for our enrichment analysis later, so we did not explore this potential bias further. The resulting dataset, consisting of 5536 genomes, will be the dataset we refer to for the rest of the paper, unless otherwise indicated.

### Protein sequences prediction and clustering

Prodigal v2.6.3 [12] was used in normal mode with default parameters to predict open reading frames and their corresponding protein translations for all genomes in the dataset. Next, CD-HIT v4.8.1 [15] was used to cluster the predicted protein sequences. The clustering identity threshold was set at 90%, based on the assumption that proteins with this sequence similarity would likely share the same function. Consequently, proteins within each cluster were considered functionally equivalent for the purposes of this study and the centroid sequence of each cluster was used as the representative sequence of the cluster in all downstream analyses.

### Identification of clusters enriched with human-derived sequences

To identify over-represented clusters among the clusters containing sequences of > 90% identity, Fisher's exact test was applied to detect clusters with an over-representation of sequences derived from human isolates. The statistical analysis was performed using the scipy Python package v1.7.3 [30] on three pairs of isolation sources: Human vs. Natural Environment, Human vs. Built Environment, and Human vs. Other.

To account for multiple testing, the p-values obtained from Fisher's exact test were adjusted using the Benjamini–Hochberg correction method from the *statsmodels* Python package v0.13.5 [27]. To identify proteins likely to be involved in human host colonization and potentially virulence, we focused our subsequent analysis solely on the results from the Human vs. Natural Environment pair test.

### Functional characterization

**Fig. 3:**
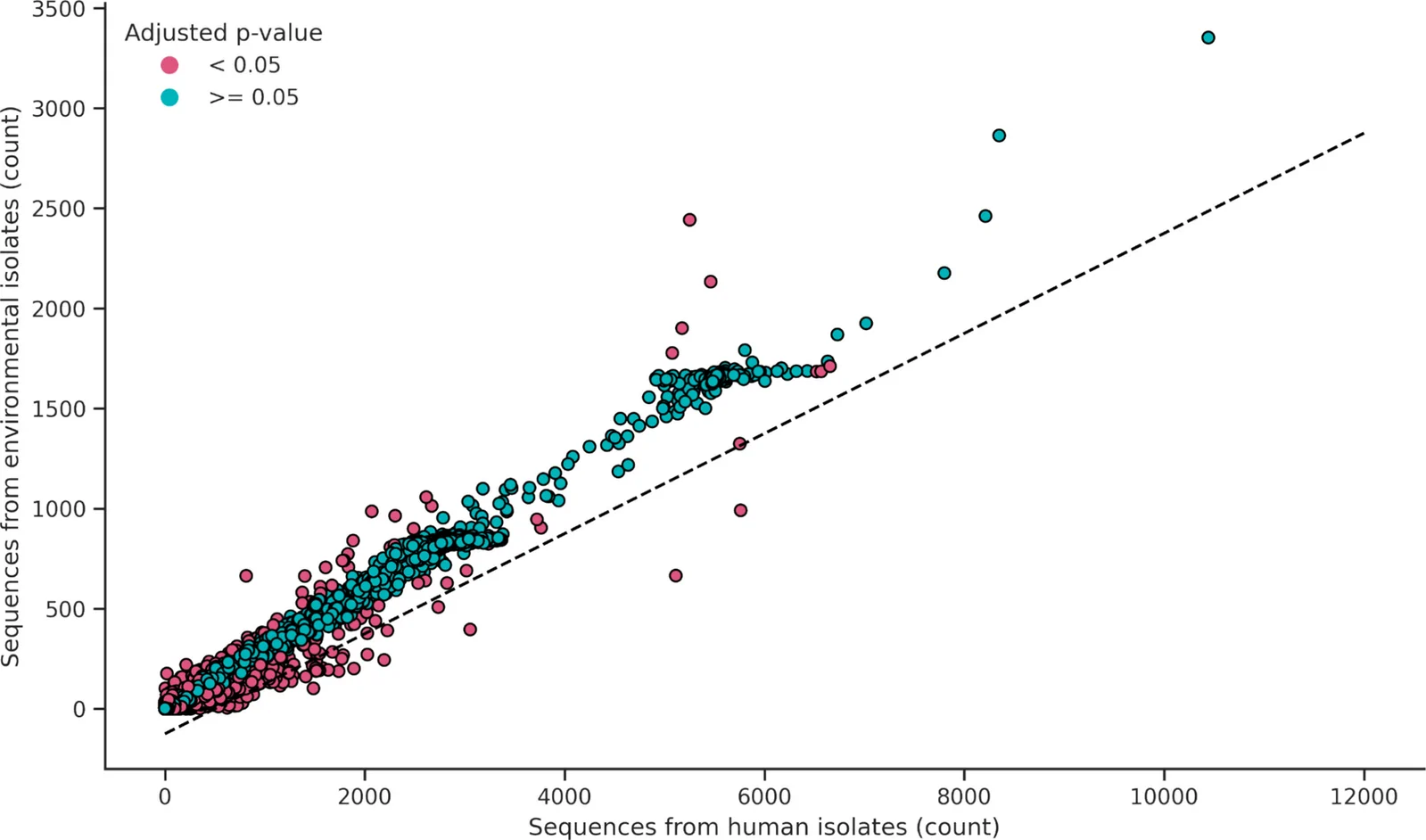
Significant clusters scatterplot. Each point represents a distinct cluster generated by CD-HIT, the x-axis shows the number of sequences from human-derived isolates and the y-axis shows the number of sequences from natural environment-derived isolates. Clusters colored in pink indicate a significant (Fisher’s corrected p-value < 0.05) over-representation of either human-derived (clusters closer to the x-axis) or natural environment-derived sequences (clusters closer to the y-axis). The dashed line represents the selection boundary used to select the subset of clusters for the functional characterization (clusters falling below the selection boundary line)

To identify the most promising candidate genes responsible for host-adaption from the Human vs. Natural Environment results, we implemented a filtering approach based on the following criteria: (1) Cluster size. Each selected cluster had to contain at least 500 Human-labeled sequences, to ensure a robust sample size. (2) Statistical significance. All chosen clusters must demonstrate statistically significant overrepresentation of sequences from human isolates, avoiding the inclusion of non-significant results. (3) Dataset size: The resulting subset should be of a manageable size to allow for thorough functional characterization and manual curation of the results. These criteria were then mathematically integrated into a selection boundary defined by the equation y = 1/4x – 125 (Fig. 3). This boundary effectively created a refined subset of 45 clusters enriched in human-associated *P. aeruginosa* genomes, which were then subjected to functional characterization using a multi-tool approach. SignalP v6.0 [28] was used in slow sequential mode with default settings to predict signal peptides.

Protein sequences were searched against the ClusteredNR database using BLASTP v2.17.0 + (S. [1]. The best hit for each protein was retained. When the top hit did not correspond to *P. aeruginosa*, the highest-ranking *P. aeruginosa* match was also retrieved if it was present within the top 20 hits. Functional characterization was further supported by conserved domain analysis. For proteins associated with a locus tag or gene name, the Pseudomonas Genome Database [32] was used to further refine functional annotations.

To determine how prevalent the selected sequences were among *P. aeruginosa* isolates, they were mapped to an in-house *P. aeruginosa* pangenome catalog (File S1), using a minimum percent identity of 80% and retaining only the top hit for each query. Finally, BLAST v2.12.0 (S. F. [2] was used to align the predicted gene sequences against the PAO1 reference genome (GCF_000006765.1) to check for overlap with known gene names or locus tags. Results were filtered to keep only the first hit with at least 90% identity.

## Results

### Accounting for the overrepresentation of human-associated isolates in public P. aeruginosa genome assemblies

**Fig. 4:**
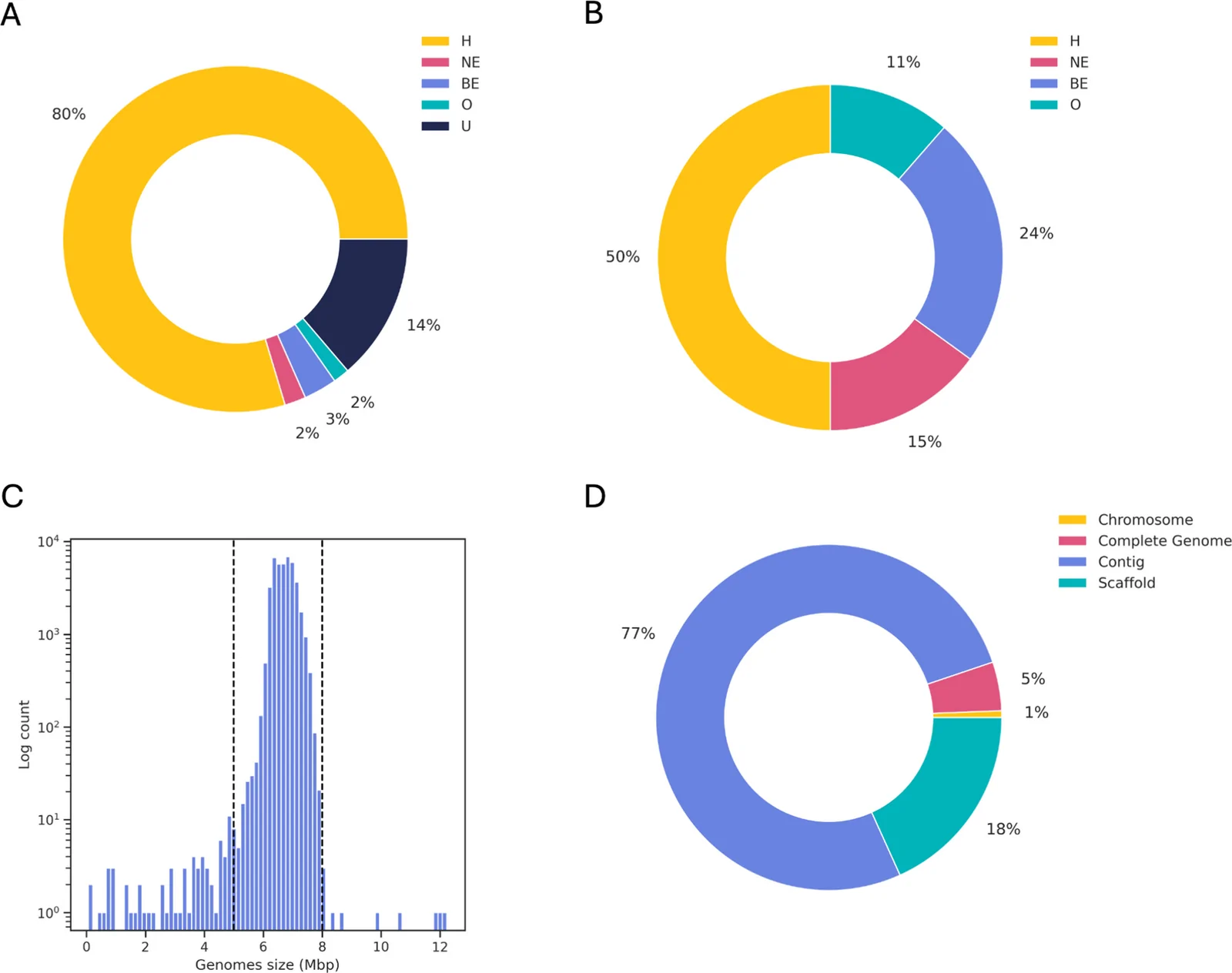
Overview of the P. aeruginosa genomes dataset. **A**. Percentage of the genomes in each category (H = human, NE = natural environment, BE = built environment, O = other, U = unknown) in the initial dataset (**A**) and the study dataset (**B**). Genome sizes (Mbp) distribution in the initial dataset, before size filtering. The vertical dashed lines correspond to the upper and lower boundaries used in the genome size filtering step (Methods 2.2). **C**. Assembly levels of the genomes in the initial dataset

The classification of the isolation sources of the 41,874 *P. aeruginosa* genomes in the initial dataset revealed a substantial bias towards human isolates (Fig. 4A). After filtering the genomes by size, 41,794 genomes were retained; discarding samples with mismatching labels further reduced the size of the dataset to 36,045 genomes. Finally, after subsampling the human isolates group, the dataset comprised 5,536 samples (Fig. 4B). Due to the subsampling method (Methods 2.2), 50% of the isolates in the final dataset came from human isolates. Isolates from built environments constituted 24%, those from natural environments represented 15%, and isolates from other hosts constituted the remaining 11%. Most of the genomes were assembled at the contig (79%) and scaffold (16%) levels, with complete genomes and chromosomes constituting only 4% and 1% of the dataset, respectively (Fig. 4D).

After size-filtering, the sizes of the genomes in our dataset ranged from 5.08 Mbp to 7.99 Mbp, with an average size of 6.68 Mbp (Fig. 4C). When compared to the *P. aeruginosa* PAO1 reference genome, which has a size of approximately 6.3 Mbp (24), this range suggests that the genomes in our dataset are sufficiently complete, while still retaining the natural variation within the *P. aeruginosa* species. It is important to note that during the size-filtering step (*Methods*), the maximum and minimum allowable genome sizes were set to 8 Mbp and 5 Mbp, respectively. 

### Identification of protein clusters overrepresented in human-associated genomes

Noting that for colonization and virulence factors to be fixed in a bacterial population, they would need to be of advantage in a host, and as a result of long-term within-host selection they would be expected to be over-represented in genomes derived from strains with a host-associated lifestyle. While environmental settings are important for the acquisition of new functions, these functions would only be enriched in a host-associated setting if they are truly beneficial in that context. We therefore next focused our analysis on comparison of the isolates from human and natural environments, since we considered this comparison to be the most likely to show differences related to virulence and the ability to colonize human hosts. We calculated the expected ratio of human isolates to natural environment isolates by dividing the total number of sequences from human isolates (nH = 2768) by the total number of sequences from natural environment isolates (nEN = 832), which served as the baseline for comparing the proportion of sequences from the two isolation sources within each cluster. 

Our findings revealed that 6% of the clusters significantly deviated from this expected ratio (corrected p-value < 0.05). About half of these significant clusters (3154) had a human to natural environment isolate ratio larger than the expected ratio (nH/nEN > 3.327), which indicates a notable enrichment of sequences from human isolates compared to environmental isolates.

### Selection of human-enriched protein clusters for downstream analysis

**Fig. 5:**
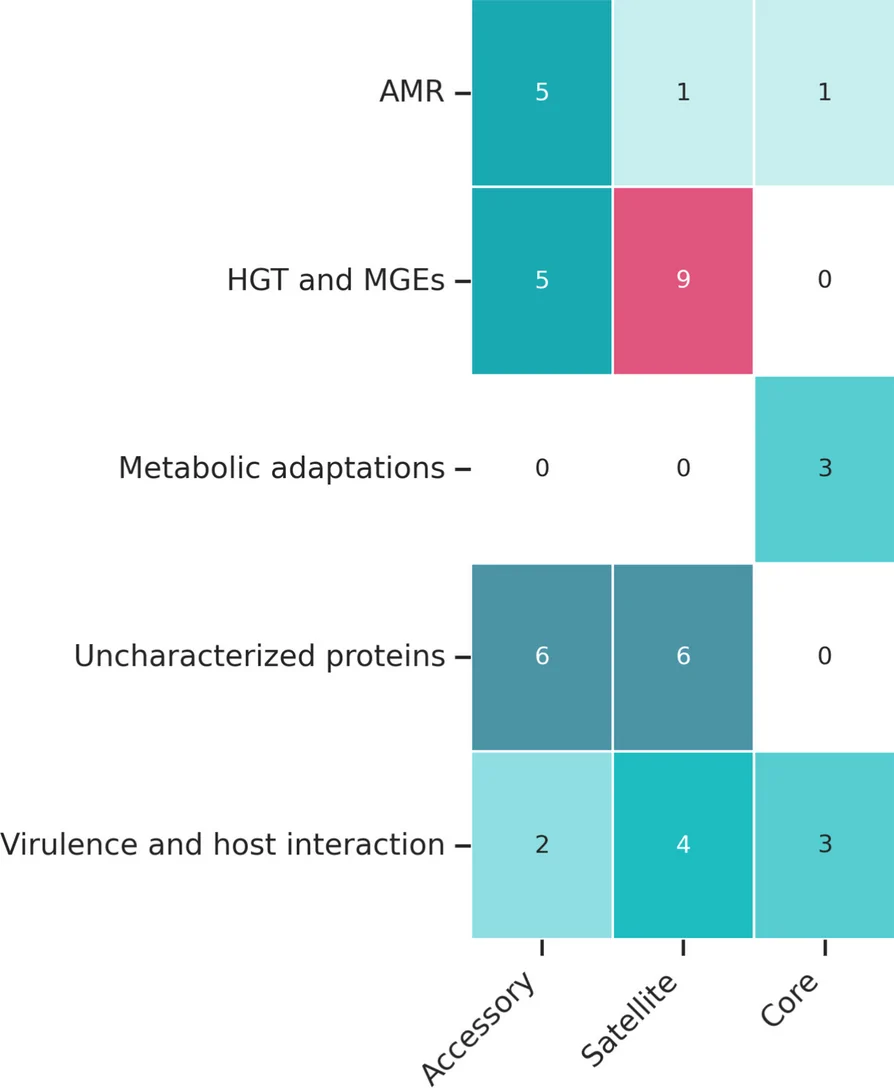
Distribution of enriched protein clusters across functional categories and pangenome compartments. Each cell displays the number of protein clusters belonging to a given functional category (y-axis) and pangenome compartment (x-axis)

To focus the analysis on biologically meaningful candidates, we selected protein clusters containing at least 500 sequences from human isolates and showing statistically significant enrichment of sequences from human isolates. This yielded 45 clusters whose centroid sequences were functionally characterized (Fig. 5). To determine the function of these proteins, we used SignalP [28] to predict the presence of signal peptides and mapped the protein sequences to the *P. aeruginosa* pangenome catalogue (File S1). Finally, blastP [1] was used to determine protein function via similarity to other *P. aeruginosa* proteins and to find conserved protein domains. The Pseudomonas Genome Database [32] was used to refine the functional characterization.

### Distribution of enriched protein clusters across pangenome compartments

Mapping 45 selected protein sequences onto the *P. aeruginosa* pangenome catalog revealed that most belong to the accessory or satellite genome. Satellite genes (20 sequences) represented the largest group, closely followed by the accessory genome (18 sequences). This distribution points to horizontal gene transfer and strain-level adaptation as primary drivers of human host specialization. Only 7 sequences were mapped to the core genome, reflecting the substantial overrepresentation of human-associated isolates among the genomes used to construct the pangenome catalog. This bias is a result of the overrepresentation of human-associated *P. aeruginosa* strains in genetic databases; including the catalogue of complete genomes available in the Pseudomonas Genome Database [32], which were used to construct the gene catalog.

### Functional characterization of human-enriched protein clusters

Functional characterization organized the 45 proteins into five categories: virulence and host interaction, horizontal gene transfer and mobile genetic elements, antimicrobial resistance, metabolic adaptation, and proteins of unknown function. Together, these proteins describe how *P. aeruginosa* colonizes and persists in the human host by resisting clearance, acquiring new capabilities and tuning its metabolism to survive in human hosts (Table 1).

**Table 1:** Functional characterization of human-enriched protein clusters

Clustering			Functional characterization			PAO1			blastP	
Cluster ID	Centroid ID	p-value	Pangenome	SignalP	Group	Locus tag	Gene name	Cellular localization	Sequence ID	Description
1	EN_KI519317.1_42	2.34e-12	Satellite	SP	Virulence and host interaction	PA0041	not found	extracellular	WP_250321820	DUF637 domain- containing protein, partial [Pseudomonas aeruginosa]
2	EN_KI519316.1_1903	2.34e-12	Satellite	OTHER	Virulence and host interaction	PA2462	not found	outer membrane	WP_034055838	hemagglutinin repeatcontaining protein, partial [Pseudomonas aeruginosa]
3	H_NZ_KI914515.1_2256	2.49e-13	Satellite	OTHER	Virulence and host interaction	PA1874	not found	unknown	WP_049885648	Ig-like domaincontaining protein, partial [Pseudomonas aeruginosa]
4	EN_KI519298.1_79	8.02e-18	Satellite	SP	Virulence and host interaction	PA4625	cdrA	extracellular	WP_423353592	MBG domain-containing protein [Pseudomonas aeruginosa]
5	EN_KI518716.1_3544	4.11e-36	Core	OTHER	Virulence and host interaction	PA1903	phzE2	cytoplasmic	MGU1470093	anthranilate synthase family protein [Pseudomonas aeruginosa]
6	EN_KI518737.1_526	4.47e-33	Core	OTHER	Virulence and host interaction	PA1902	phzD2	unknown	WP_250866310	phenazine biosynthesis protein PhzD, partial [Pseudomonas aeruginosa]
7	H_CABISD010000007.1_264	4.58e-27	Satellite	OTHER	HGT and MGEs	not found	not found	not found	WP_335929620	phage tail tape measure protein, partial [Pseudomonas aeruginosa]
8	H_DAIJSQ010000002.1_121	4.38e-23	Accessory	OTHER	AMR	PA3690	not found	cytoplasmic membrane	HCF0794478	lipoprotein signal peptidase [Pseudomonas aeruginosa]
9	H_LCSQ01000262.1_31	1.02e-34	Accessory	OTHER	HGT and MGEs	not found	TraD	not found	WP_132782154	type IV conjugative transfer system coupling protein TraD [Pseudomonas aeruginosa]
10	H_NZ_NIOU01000033.1_137	1.77e-23	Satellite	OTHER	HGT and MGEs	not found	not found	not found	WP_108232674	MULTISPECIES: conjugative transfer ATPase [Pseudomonadaceae]
11	H_DANLUG010000009.1_121	2.51e-19	Satellite	OTHER	HGT and MGEs	not found	not found	not found	CDI95045	conjugal transfer ATPase TrbE [Pseudomonas aeruginosa PA38182]
12	EN_NZ_NMRS01000008.1_774	3.59e-14	Accessory	OTHER	Virulence andhost interaction	PA0262	vgrG2b	cytoplasmic	WP_319122579	type VI secretion system host interaction spike protein VgrG2a
13	H_JAPCST010000050.1_113	2.40e-10	Accessory	OTHER	Virulence and host interaction	PA5266	vgrG6	cytoplasmic	WP_126869835	type VI secretion system Vgr family protein [Pseudomonas aeruginosa]
14	EB_CP133416.1_1774	6.36e-45	Core	OTHER	Metabolic adaptations	PA2040	pauA4	cytoplasmic	MGU2463567	glutamine synthetase family protein [Pseudomonas aeruginosa]
15	EN_NZ_JAJEJW010000001.1_57	3.15e-37	Core	OTHER	Virulence and host interaction	PA0095	vgrG1b	cytoplasmic	WP_119519327	type VI secretion system spike protein VgrG1b [Pseudomonas aeruginosa]
16	O_NZ_RAFM01000369.1_24	1.80e-20	Accessory	OTHER	HGT and MGEs	not found	not found	not found	WP_168334285	helicase-related protein [Pseudomonas aeruginosa]
17	H_JAOVBS010000003.1_193	6.42e-26	Accessory	OTHER	HGT and MGEs	not found	not found	not found	WP_022580073	conjugal transfer protein TraG N-terminal domaincontaining protein [Pseudomonas aeruginosa]
18	EB_NZ_RHTB01000063.1_14	3.77e-27	Satellite	OTHER	HGT and MGEs	not found	not found	not found	HBO7541001	TIGR03749 family integrating conjugative element protein [Pseudomonas aeruginosa]
19	H_DAHOXF010000091.1_1	1.90e-16	Satellite	OTHER	Uncharacterized proteins	not found	not found	not found	MDF5961162	hypothetical protein [Pseudomonas aeruginosa]
20	EB_NZ_JBBNBK010000003.1_2662	3.54e-25	Satellite	OTHER	HGT and MGEs	PA0091	tadA	extracellular	STU23969	hypothetical protein [Pseudomonas aeruginosa]
21	EB_NZ_JAFEUG010000003.1_126	1.96e-52	Accessory	SP	AMR	PA2296	not found	unknown	MBH4297464	ABC transporter substrate-binding protein [Pseudomonas aeruginosa]
22	EB_NZ_JAFEUG010000020.1_180	1.62e-48	Core	OTHER	Metabolic adaptations	PA0439	not found	cytoplasmic	WP_263915919	NAD-dependent dihydropyrimidine dehydrogenase subunit PreA [Pseudomonas aeruginosa]
23	EB_NZ_LNIV02000002.1_104	7.09e-50	Core	OTHER	Metabolic adaptations	PA1555	ccoP2	unknown	MGU1549460	cbb3-type cytochrome c oxidase subunit I [Pseudomonas aeruginosa]
24	H_NZ_JAGFDP010000005.1_146	3.32e-24	Satellite	OTHER	HGT and MGEs	not found	not found	not found	WP_282414940	TIGR03752 family integrating conjugative element protein [Pseudomonas aeruginosa]
25	H_NZ_JAMOGZ010000002.1_829	1.10e-17	Satellite	SP	HGT and MGEs	not found	not found	not found	OKN81321	integrating conjugative element protein [Pseudomonas aeruginosa]
26	H_NZ_LEJT01000012.1_308	2.94e-43	Satellite	OTHER	AMR	PA1848	not found	cytoplasmic membrane	WP_423740040	MFS transporter [Enterobacter kobei]
27	EB_NZ_LSZT01000006.1_37	1.66e-19	Satellite	OTHER	Uncharacterized proteins	PA0256	not found	cytoplasmic	WP_326852474	DUF6094 domaincontaining protein [Burkholderia cenocepacia]
28	H_JAEIER010000047.1_30	2.30e-49	Core	LIPO	AMR	PA3214	not found	unknown	WP_319061140	ABC-type transport auxiliary lipoprotein family protein [Pseudomonas aeruginosa]
29	H_NZ_QJPG01000228.1_10	6.38e-41	Accessory	OTHER	AMR	PA4750	folP	cytoplasmic	XNY81299	Dihydropteroate synthase type-2 Sulfonamide resistance protein [Providencia rettgeri]
30	H_JAUAWJ010000008.1_52	1.19e-25	Accessory	OTHER	Uncharacterized proteins	not found	not found	not found	WP_234852126	DUF6629 family protein [Pseudomonas aeruginosa]
31	O_NZ_RAFW01000026.1_5	6.46e-19	Satellite	OTHER	HGT and MGEs	PA5563	soj	cytoplasmic membrane	WP_164080517	ParA family protein [Stenotrophomonas maltophilia]
32	H_CAULEU010000057.1_36	1.38e-27	Satellite	OTHER	Uncharacterized proteins	not found	not found	not found	WP_238170609	gp436 family protein [Pseudomonas aeruginosa]
33	EN_LXFB01000048.1_1	1.81e-12	Accessory	OTHER	Uncharacterized proteins	not found	not found	not found	RTB58471	DUF3164 family protein [Pseudomonas aeruginosa]
34	EB_NZ_JBBNBK010000004.1_46	1.46e-23	Accessory	OTHER	Uncharacterized proteins	not found	not found	not found	WP_340627106	hypothetical protein [Pseudomonas aeruginosa]
35	H_NZ_LCSV01000013.1_45	3.97e-19	Satellite	OTHER	Uncharacterized proteins	not found	not found	not found	CDF84551	hypothetical protein PKB_3206 [Pseudomonas knackmussii B13]
36	H_NZ_JNHD01000019.1_76	1.20e-16	Accessory	OTHER	HGT and MGEs	PA5563	soj	Cytoplasmic membrane	WP_289239165	ParA family partition membrane ATPase [Pseudomonas
37	H_NZ_JAWDQD010000002.1_692	2.43e-23	Accessory	SP	Uncharacterized proteins	not found	not found	not found	VCZ00663	hypothetical protein BANRA_04234 [Pseudomonas aeruginosa]
38	H_NZ_QVBC02000149.1_1	4.15e-20	Accessory	OTHER	Uncharacterized proteins	not found	not found	not found	WP_271013839	DUF2857 domaincontaining protein [Pseudomonas aeruginosa]
39	EN_NZ_NMRS01000010.1_789	4.65e-23	Satellite	OTHER	HGT and MGEs	not found	not found	not found	WP_261659709	MULTISPECIES: TIGR03750 family conjugal transfer protein [Cupriavidus]
40	H_JAVFCZ010000204.1_1	1.59e-18	Satellite	OTHER	Uncharacterized proteins	not found	not found	not found	WP_475559325	hypothetical protein [Aeromonas caviae]
41	H_ABFHHZ010000475.1_1	4.84e-25	Accessory	OTHER	AMR	not found	not found	not found	WLW16213	inner membrane transporter protein [uncultured bacterium]
42	H_DARSAS010000002.1_144	1.92e-23	Accessory	SP	HGT and MGEs	not found	not found	not found	OYZ46314	MAG: conjugal transfer protein [Acidovorax sp. 16–64–162]
43	EB_NZ_NRAF01000084.1_3	1.78e-28	Accessory	SP	AMR	not found	not found	not found	WP_134516222	mercury resistance system periplasmic binding protein MerP, partial [Pseudomonas aeruginosa]
44	EN_NZ_LLLV01000001.1_573	3.48e-15	Accessory	OTHER	Uncharacterized proteins	not found	not found	not found	WP_013520766	MULTISPECIES: hypothetical protein [Pseudomonadota]
45	H_JADIQW010000011.1_1	5.46e-15	Satellite	OTHER	Uncharacterized proteins	not found	not found	not found	WP_256825273	DUF7364 domaincontaining protein, partial [Pseudomonas aeruginosa]

### Virulence and Host Interaction

This category consists of proteins involved in direct interaction with host tissues and includes adhesion-related proteins, proteins forming the Type VI secretion system (T6SS) and proteins involved in the biosynthesis of phenazine. Hemagglutinins and adhesion-related proteins facilitate attachment to host tissues. Both proteins falling into this group (PA0041 and PA2462) also include signal peptides, consistent with a secreted or surface-exposed role in host attachment. Once inside the host, the bacterium must establish and maintain a persistent niche. The two-partner secretion system (PA4625) facilitates this process by mediating the interactions with the host environment through the secretion of large extracellular proteins [8].

To compete and survive within the host environment, *P. aeruginosa* utilizes the type VI secretion system (T6SS) to inject toxic effector proteins into neighboring bacteria and host cells [9]. We identified three different VgrG spike proteins being enriched in the human-associated isolates: VgrG1b (PA0095), VgrG2b (PA0262), and VgrG6 (PA5266). These are all key structural components of the apparatus that facilitate effector delivery to target cells [10]. Finally, the phenazine biosynthesis pathway (PA1903 and PA1902) produces redox-active metabolites that support anaerobic survival within biofilms and antibiotic tolerance while simultaneously generating reactive oxygen species that damage host tissue [24].

### Antimicrobial Resistance

Survival in the human host requires tolerance not only to immune defenses but also to the stresses imposed by the use of antibiotics in clinical settings. Many of the proteins analyzed were found to be involved in processes related to antibiotic resistance and stress response mechanisms. MerP and MerT, key components of the *mer* operon which confers resistance to mercury, are frequently co-located on mobile genetic elements with antibiotic resistance determinants [19], making their enrichment a possible proxy for the broader resistance gene carriage. Dihydropteroate synthase (PA4750), the target of sulfonamide antibiotics [26], was also among the protein enriched in human isolates. Resistant variants of this enzyme are commonly found on plasmids and maintain folate biosynthesis by evading drug binding. Their presence in human-enriched clusters is consistent with clinical antibiotic pressures shaping the *P. aeruginosa* population [29].

### Metabolic Adaptations

Chronic infection presents several metabolic challenges for *P. aeruginosa*, including those related to fluctuating oxygen levels and nutrient limitation. The cbb3-type cytochrome c oxidase subunit (PA1555) addresses the oxygen problem: unlike conventional terminal oxidases, this enzyme has exceptionally high oxygen affinity, sustaining aerobic respiration even in the microaerobic conditions of biofilms and mucus [11]. Furthermore, gutamylpolyamine synthetase (PA2040) contributes to polyamine metabolism, while NAD-dependent dihydropyrimidine dehydrogenase (PA0439) participates in pyrimidine catabolism. Both these enzymes likely support growth under unstable and nutrient-poor conditions that characterize human tissues. Finally, since metal scarcity is a key feature of the human host environment, proteins related to the transport of metal ions (P-type ATPase (PA3690) and ABC transporters (PA2296 and PA3214)) likely contribute to P. aeruginosa’s survival under metal-limited conditions [17].

### Horizontal gene transfer and mobile genetic elements

A striking feature of the set of proteins we analyzed is the prominence of machinery for acquiring and integrating foreign DNA. Multiple components of integrating conjugative elements (ICEs) were identified, including conjugative transfer ATPases (TraD, TraG, and TrbE), ICE-associated proteins and additional conjugal transfer components. The presence of these proteins suggests that horizontal gene transfer plays a role in *P. aeruginosa*’s adaptation to human hosts. Several phage-related proteins were also identified. However, these may represent components of the T6SS rather than, or in addition to, prophage elements. This distinction is important because the T6SS contractile apparatus is evolutionarily derived from contractile bacteriophage tails [14]. Consequently, many T6SS structural proteins, including the tail tube, sheath, and spike components, share substantial sequence and structural similarity with phage tail proteins, making them difficult to distinguish based on sequence homology alone.

### Uncharacterized proteins

Twelve proteins remained uncharacterized despite efforts to structurally infer function via Alphafold and FoldSeek. Of these, 6 proteins carry domains of unknown function (DUF6094, DUF6629, DUF3164, DUF2857, DUF7364, DUF1320) and 6 are hypothetical proteins. All proteins belong to the accessory or satellite genome and only one is predicted to contain a signal peptide. The significant enrichment of these proteins in human isolates suggests a potential to hold important, but currently unrecognized, roles in human colonization. Therefore, their characterization represents a priority for future work.

## Discussion

In this study we used a comparative genomics approach to identify the traits that distinguish *P. aeruginosa* strains capable of colonizing and persisting in the human body from strains isolated from natural environment settings. We focused our analysis on 45 clusters consisting of proteins significantly overrepresented in human isolates, revealing a coherent set of biological strategies involved in human colonization. These span tissue attachment, chronic infection establishment, metabolic flexibility and the capacity to rapidly acquire new traits. The functional signatures identified in this study align well with established understanding of *P. aeruginosa* virulence and host interaction, suggesting that a population-level comparative genomics approach can uncover true biological strategies that contribute to pathogenesis and colonization success.

The predominance of mobile genetic elements and conjugative transfer machinery among the overrepresented proteins indicates that horizontal gene transfer is a crucial mechanism for acquiring host-adaptive traits. This aligns with our broader understanding of bacterial evolution in clinical settings, where genetic exchange accelerates adaptation and enables rapid spread of advantageous traits through bacterial populations.

The distribution of overrepresented proteins across the pangenome provides additional important context. The large number of overrepresented proteins in satellite and accessory genome categories indicates that success in human hosts depends heavily on acquired genetic material and that adaptation to a human-associated lifestyle is a dynamic process. These findings are consistent with previous studies by Berger et al. [4] and Freschi et al. [7], which demonstrated the central role of the accessory genome in the pathogenicity and host adaptation of *P. aeruginosa*. This highlights the importance of acquired genetic elements in facilitating adaptation to human hosts. The few core genes that do appear overrepresented likely reflect a technical artifact. This is because the completely sequenced *P. aeruginosa* genomes used to construct the pangenome catalogue have a bias towards human-associated isolates. Therefore, if a gene is systematically missing in environmental strains, it may still be considered “core” by the genome catalog due to being omnipresent in human-associated isolates. This suggests that we might either want to include a more diverse set of *P. aeruginosa* genomes in the genome catalogs, or look differently at environment- or context-specific cores rather than referring to the core genome of the entire species, as ecotypes might have diverged substantially in terms of core functionality.

This study simultaneously also suggests that current knowledge provides only a partial explanation for the complete protein landscape associated with human host colonization, since 24.5% of the significantly enriched proteins in human-associated strains remained uncharacterized. The fact that these proteins have been maintained and enriched implies functional importance and that they provide selective advantages during human colonization and/or infection. That said, experimental validation of the genes with unknown function identified in this study would be necessary to verify which of these are actually involved in host-adaptation and which might have the potential to be virulence factors.

An important insight emerges when comparing this genomic enrichment approach to other methodologies for identifying genes involved in host-adaptation. A previous large-scale mutation-based analysis (2024) employed a complementary strategy to the one presented here, systematically disrupting individual genes in laboratory strains and assessing effects on pathogenesis in infection models. Remarkably, only two of the 45 genes identified in the present study (PA0439 and PA3214) were recovered in that mutational screen.

This minimal overlap highlights the complementary nature of different methodological approaches, where the mutational analysis approach excels at identifying genes whose disruption causes observable, often dramatic, effects on infection phenotypes in controlled laboratory settings, while the genomic enrichment approach employed here identifies genes that have been preferentially retained and confer selective advantages in real the human host. The analysis of naturally occurring *P. aeruginosa* isolates compared to human-associated ones captures the genetic signatures of strains that have actually succeeded in human colonization, without prior selection of presumably relevant targets.

To further validate our candidate genes, we cross-checked our results with two independent studies that compared *P. aeruginosa* transcriptional profiles under conditions relevant to host adaptation (Table S1). Cornforth et al. [6] compared the transcriptome of *P. aeruginosa* during human infection with that of bacteria grown under a range of laboratory conditions, thereby identifying transcriptional changes associated with the transition to the host environment. Rossi et al. [21] similarly compared *P. aeruginosa* transcriptomes from human infection with those of bacteria grown in vitro to characterize transcriptional changes associated with the pathophysiological host environment. Approximately half of our candidate proteins were significantly differentially expressed in the Cornforth et al. study, while two overlapped with the candidates identified by Rossi et al. The significant differential expression of several of our candidate genes across independent studies provides supporting evidence for their potential involvement in host adaptation or virulence. Importantly, genes involved in host adaptation do not necessarily need to be upregulated in the host relative to laboratory conditions,rather, their expression may simply need to be maintained under host-associated conditions. Therefore, the direction of differential expression is not necessarily the most relevant criterion when assessing their potential role in host adaptation.

Taken together, the genomic and transcriptomic analyses provide multiple complementary lines of evidence for the candidate genes identified in this study. To facilitate the interpretation and prioritization of the candidate genes for future studies, we compiled an evidence-based matrix integrating transcriptomic evidence, functional characterization, cellular localization and genomic-context information (Table S2). This is intended as a guide to the selection of candidates for future experimental validation, rather than to establish a causal role in human-host adaptation. For this reason, we present the characteristics of each candidate separately to provide readers with a transparent basis for evaluating the candidates according to their own research interests and priorities. We suggest that particular attention may be given to candidates supported by transcriptomic evidence and functional annotations related to virulence, host interaction, or metabolic adaptation, especially when these features are accompanied by predicted extracellular or outer-membrane localization and/or a signal peptide. Additionally, candidates with uncharacterized functions should also be considered, as the absence of an assigned function may reflect genes that have not yet been characterized and could represent novel mechanisms associated with human-derived isolates.

Relying on publicly available data comes with limitations that might affect the completeness and accuracy of findings. Publicly available genome sequences often come with varying quality of the associated metadata regarding isolation source and host characteristics. While we implemented a conservative genome classification procedure, the possibility of errors remains. Additionally, the inherent bias in favor of human-associated strains in the initial dataset could have affected our ability to fully capture the genetic diversity of *P. aeruginosa.* Although we employed subsampling to mitigate the imbalance, future studies would benefit from broader environmental strain representation and more standardized metadata.

Despite these limitations, the consistency of several of our findings with established knowledge of *P. aeruginosa* virulence and host colonization, together with the functional coherence of the majority of our enriched gene clusters, suggests that the patterns identified reflect mechanisms involved in host-adaptation. The comparative genomics framework developed in this study therefore serves as a first step toward identifying genes associated with human-derived isolates and as a tool to prioritize candidates for experimental investigation. The method is also not limited to *P. aeruginosa* and could be applied to study less well-characterized organisms where such candidates may provide new insights into host-associated adaptation.

## Supplementary Information

Supplementary Material 1

Supplementary Material 2

Supplementary Material 3

Supplementary Material 4

## Data Availability

No new experimental data were generated in this study. All data used were obtained from the National Center for Biotechnology Information (NCBI) Assembly database, the list of the accession numbers of the genomes used in this study is available in the supplementary material (file S1). The Pseudomonas aeruginosa genome used as reference genome is NCBI RefSeq assembly GCF_000006765.1. The code used for the analysis is available at https://github.com/tctat13/pa-host-adaptation/.
