# Supplementary material for: On the hunt for candidate genes involved in host-adaptation in Pseudomonas aeruginosa using comparative genomics
Source: BMC Genomics. 2026 Oct 3;27:815. doi: 10.1186/s12864-026-13405-3 (PMC13637380; doi:10.1186/s12864-026-13405-3)
Supplement: Supplementary file 1 [file 12864_2026_13405_MOESM1_ESM.docx]

**Supplementary Method**

***Pseudomonas aeruginosa* pangenome catalog**

On 15 April 2023, 494 complete genomes available on the *P. aeruginosa* were downloaded from the Pseudomonas Genome Database [1] in nucleotide sequence, amino acid sequence and GFF formats. The amino acid sequence files were processed with in-house Python scripts to relabel the FASTA headers to unique 16-character alphanumeric codes for ease of processing. The amino acid FASTAs for the complete genomes were clustered at 95% identity using CD-HIT version 4.8.1 [2,3] with the following flags: *-c 0.95 -n 5 -M 1000000 -T 64 -p 1 -g 1*

Protein sequences clustered via this 95% identity threshold were considered to be the same protein for our purposes and will be referred to as genes throughout the rest of this text. The identified proteins were used to create a pangenome catalog consisting of the following categories of proteins, based on how consistently they appear across complete genomes: core genome, accessory genome, satellite genome, singletons and paralog only (Table 1). If multiple copies of a gene were identified, but all copies originate from the same complete genome, these were categorized as “paralog only.” As some of these may be technical artifacts resulting from issues with sequencing, we decided not to include these clusters within the satellite category. The results of this categorization are presented in Figure 1. Based on our pangenome catalog category definitions, around 5000 genes were identified as belonging to the core genome. The vast majority of genes were categorized as belonging to either the satellite or singleton categories.

In-house scripts were used to combine existing data into a dataframe containing information on the gene, pangenome catalog category, gene copy identifiers, and gene copy sequences; as well as to output FASTA files containing the sequence information on all copies of each identified gene. Multiple sequence alignments of these gene FASTA files were created using MAFFT version 7.520 [4,5]. These MSAs were used to create consensus sequences using Emboss Cons from the EMBOSS package version 6.6.0 [6,7].

Command line BLAST version 2.15.0 [8,9] was used to generate BLAST databases from the consensus sequences and representative sequences generated by CD-HIT during clustering. These BLAST databases, along with BLAST functionality and an in-house BLAST result parsing script were packaged into the pa-BLAST.sif Apptainer container created with Apptainer version 1.2.5 [10,11]. Using this container enabled BLAST-ing against the *P. aeruginosa* pangenome catalog, allowing for comparisons with other genes and data used in this analysis.

The initial FASTA file parsing was done with Python version 3.9.16 in Spyder version 5.4.3 [12,13]. All subsequent data processing and file restructuring was done with Python version 3.9.18 in Spyder version 5.4.5 [12,13]. Visualization of the pangenome catalog was performed with R version 4.3.2 in RStudio version 2023.12.0+369 using ggplot2 [14–16].

***Table S1: Definitions of pangenome catalog categories used, based on percentage of complete genomes the gene was identified in****.*

| **Category** | **Definition** |
| --- | --- |
| Core | Gene present in >95% of complete genomes. |
| Accessory | Gene present in 10%-95% of complete genomes. |
| Satellite | Gene present in <10% of genomes. |
| Singleton | Only one copy of the gene identified across all studied genomes. |
| Paralog only | Gene identified multiple times, but all copies originate from the same complete genome. |

***Figure S1: Number of genes present in each frequency category on the pangenome catalog****. Information on the definitions of the pangenome catalog categories is provided in Table 1.*

**Figure S2: Average nucleotide identity (ANI) between all pairs of genomes from the same isolation source in the subsampled dataset.**
The ANI was calculated using used skani (v0.3.0) as follows:
./skani sketch dataset_subsample/*.fna -o skani_index

./skani search -t 40 -d skani_index dataset_subsample/*.fna > skani_hits.tsv

**References:**

1.	Winsor GL, Griffiths EJ, Lo R, Dhillon BK, Shay JA, Brinkman FSL. Enhanced annotations and features for comparing thousands of Pseudomonas genomes in the Pseudomonas genome database. Nucleic Acids Res. 2015;44: D646. doi:10.1093/NAR/GKV1227

2.	Fu L, Niu B, Zhu Z, Wu S, Li W. CD-HIT: accelerated for clustering the next-generation sequencing data. Bioinformatics. 2012;28: 3150–3152. doi:10.1093/BIOINFORMATICS/BTS565

3.	Li W, Godzik A. Cd-hit: a fast program for clustering and comparing large sets of protein or nucleotide sequences. Bioinformatics. 2006;22: 1658–1659. doi:10.1093/BIOINFORMATICS/BTL158

4.	Katoh K, Misawa K, Kuma KI, Miyata T. MAFFT: a novel method for rapid multiple sequence alignment based on fast Fourier transform. Nucleic Acids Res. 2002;30: 3059–3066. doi:10.1093/NAR/GKF436

5.	Katoh K, Standley DM. MAFFT multiple sequence alignment software version 7: Improvements in performance and usability. Mol Biol Evol. 2013;30: 772–780. doi:10.1093/molbev/mst010

6.	Rice P, Longden I, Bleasby A. EMBOSS: The European MolecularBiology Open Software Suite. Trends in Genetics. 2000;16. doi:https://doi.org/10.1016/S0168-9525(00)02024-2

7.	EMBOSS: cons. [cited 16 Dec 2024]. Available: https://emboss.sourceforge.net/apps/cvs/emboss/apps/cons.html

8.	Altschul SF, Gish W, Miller W, Myers EW, Lipman DJ. Basic local alignment search tool. J Mol Biol. 1990;215: 403–410. doi:10.1016/S0022-2836(05)80360-2

9.	Entrez Direct: E-utilities on the Unix Command Line - Entrez Programming Utilities Help - NCBI Bookshelf. [cited 18 May 2021]. Available: https://www.ncbi.nlm.nih.gov/books/NBK179288/

10.	Kurtzer GM, Sochat V, Bauer MW. Singularity: Scientific containers for mobility of compute. PLoS One. 2017;12: e0177459. doi:10.1371/JOURNAL.PONE.0177459

11.	Kurtzer GM, cclerget, Bauer M, Kaneshiro I, Trudgian D, Godlove D. hpcng/singularity: Singularity 3.7.3. Zenodo; 2021. doi:10.5281/zenodo.4667718

12.	Van Rossum G, Drake FL. Python 3 Reference Manual. Scotts Valley, CA: CreateSpace; 2009.

13.	Raybaut P. Spyder-documentation. Available online at: pythonhosted.org. 2009.

14.	R Core Team. R: A language and environment for statistical computing. R Foundation for Statistical Computing. R Foundation for Statistical Computing, Vienna, Austria; 2021. Available: https://www.r-project.org/

15.	RStudio Team. Studio: Integrated Development for R. RStudio. PBC, Boston, MA; 2020. Available: http://www.rstudio.com/

16.	Wickham H. ggplot2: Elegant Graphics for Data Analysis Second Edition. 2nd ed. New York: Springer-Verlag; 2016. Available: https://ggplot2.tidyverse.org/
